# ^18^FDG Uptake in Sinonasal Inverted Papilloma Detected by Positron Emission Tomography/Computed Tomography

**DOI:** 10.1100/2012/943412

**Published:** 2012-07-31

**Authors:** E. Allegra, M. G. Cristofaro, L. G. Cascini, N. Lombardo, O. Tamburrini, A. Garozzo

**Affiliations:** ^1^Otolaryngology Head and Neck Surgery Unit, Policlinico Germaneto-“Magna Graecia”, University of Catanzaro, 88100 Catanzaro, Italy; ^2^Maxillo-Facial Surgery Unit, Policlinico Germaneto-“Magna Graecia”, University of Catanzaro, 88100 Catanzaro, Italy; ^3^Radiology Unit, Policlinico Germaneto-“Magna Graecia”, University of Catanzaro, 88100 Catanzaro, Italy

## Abstract

Inverted papilloma (IP) is a benign but locally aggressive sinonasal tumour. Aggressive surgical treatment has thus been traditionally recommended because of the risk of transformation in squamous carcinoma. CT and MRI are used to evaluate bone destruction and soft-tissue extension before surgery but may be ineffective to differentiate an inverted papilloma from squamous cell carcinoma. In recent years, F-18 Fluorodeoxyglucose positron emission tomography (^18^FDG-PET) is widely used as diffuse imaging procedure for diagnosis and followup of malignancy affecting the head and neck district. To evaluate the utility of ^18^FDG-PET/CT in the diagnosis of patients with suspicious lesions for IP, twelve patients with suspicious sinonasal inverted papilloma were selected for this study. ^18^FDG-PET/CT imaging was performed, and standard uptake value (SUV) was calculated for each patient. SUV_max⁡_ was considered as the maximum value measured in the visualized lesions. Seven of the twelve cases exhibited uptake of ^18^FFDG with an SUV_max⁡_ ranging from 1 to 8.1. Histopathologic diagnosis after surgery confirmed the diagnosis of IP in five cases; all these cases had an SUV_max_ > 3. The five cases, which exhibited absence of ^18^FDG uptake, had a histological diagnosis of absence of IP.

## 1. Introduction

Inverted papilloma (IP) is a benign but locally aggressive sinonasal tumour that origins from the ectodermal epithelium of the nose and paranasal sinuses. Its endophytic growth results in erosion of surrounding stroma and bone. The inverted papilloma is a rare disease accounting from 0.5 to 4% of all nasal tumours [1], but its significance is related to the tendency to recur after removal. Recurrence of IP following surgery is correlated to several factors including tumor location, extent, and methods of removal, but the most important determinant of recurrence is the completeness of resection.

Aggressive surgical treatment has thus been traditionally recommended [2] because the risk of transformation in squamous carcinoma is estimated as 5–15% of cases [3].

These evidences support the use of diagnostic imaging, although the clinical evaluation is the main step in the evaluation of patients with IP. In particular CT and MRI are used to evaluate bone destruction and soft-tissue extension before surgery, but it may be ineffective to differentiate an inverted papilloma from squamous cell carcinoma [4, 5].

 In recent years, F-18 Fluorodeoxyglucose positron emission tomography (^18^FDG-PET) is widely used as diffuse imaging procedure for diagnosis and follow up of malignancy affecting the head and neck district [6].

In a previous study on patients with suspicious recurrence of IP we reported the ability of ^18^FDG-PET to detect the recurrence of IP in patients that presented a ^18^FDG-PET uptake in their lesion [7].

The purpose of this study was to evaluate the utility of ^18^FDG-PET/CT in the diagnosis of patients with suspicious lesions for IP.

## 2. Materials and Methods

### 2.1. Patients

The study has been carried out on 352 patients undergoing outpatient visit for sinonasal symptoms at the Department of Otolaryngology, Head and Neck Surgery and Maxillofacial Surgery at the University of Catanzaro, in the period between 2008 to 2010. All patients were submitted to a standard diagnostic workup composed of clinical examination and nasal endoscopy. A total of 12 patients were selected: 7 patients with suspected primary diagnosis of inverted papilloma, and 5 patients previously treated for IP with suspected recurrence of inverted papilloma. The selection criteria were: unilateral nasal obstruction, unilateral polypoid neoformation like, nasal discharge, and facial pain.

All patients included in the study provided informed consent to perform a ^18^FDG-PET/CT before surgery instead of standard computed tomography. The study was approved by the institutional review board of the “Magna Graecia” University of Catanzaro.

### 2.2. Measurements of ^18^FDG-PET Uptake

^18^FDG-PET/CT was performed before surgery. PET images (ECAT EXACT 47; Siemens) were acquired using a two-dimensional whole-body mode 60 min after the administration of 370 MBq of ^18^FDG via intravenous injection. Irregular regions of interest (ROIs) were semiautomatically drawn by the same investigator (C.G.) on transaxial planes using a dedicated workstation and software (e.soft version 4.0.8.15; Siemens). For each patient, SUV was calculated as follows: SUV = (measured activity concentration [Bq/mL])/(injected activity [Bq]/body weight [Kg] × 1,000). SUV_max⁡_  was considered as the maximum value measured in the visualized lesions. Whole-body CT was performed without the administration of intravenous or oral contrast. In this study, our series of patients ^18^FDG-PET/CT scan has been acquired by using a low-dose CT. In particular this technical approach warrants an acceptable dosimetric impact for the patient because the emission PET scan gives an effective radiation dose of 6-7 mSv irrespective of the length of acquisition.

Statistical analysis has been made using MedCalc software version 9.0. An independent *t*-test was used to compare SUV_max⁡_ between groups, test was 2-tailed, and *P* < 0.05 was considered significant.

## 3. Results

### 3.1. Patients

Eleven patients were male and one female, and their average age was 58.5 years (range, 35–73 years). They presented mainly unilateral nasal obstruction, facial pain, and rhinorrea. On physical examination using nasal endoscopy, a unilateral similpolypoid neoformation was observed in the nasal cavity in all the patients.

These patients were assessed using ^18^FDG/PET before surgical treatment. Ten of the twelve patients underwent surgical excision via functional endoscopic sinus surgery, and two patients underwent endoscopic-assisted surgery. From each patient surgical specimens were taken from the different involved localizations and separately submitted to histological examination.

### 3.2. Measurements of ^18^FDG Uptake

The ^18^FDG-PET findings of the patients and their clinical data are summarized in Table 1.

Seven of the Twelve (58.3%) patients exhibited ^18^FDG uptake. These patients showed an ^18^FDG uptake with a SUV_max⁡_ that ranged from 1 to 8.1, with a maximum SUV of 8.1 detected in a case with a histological diagnosis of recurrent IP associated with SCC.

The histological diagnosis was inverted papilloma in 5 of the 12 patients examined, while 7 were negative for IP. All patients (100%) with histological diagnosis of inverted papilloma showed ^18^FDG uptake on FDG-PET/CT images; in this group the mean SUV_max⁡_ value was of 5.88 ± 1.3 (range 4.5–8.1). Of the seven patients with negative diagnosis for IP, five (71.4%) showed no signs of the ^18^FDG uptake on FDG-PET/CT; in this group the mean SUV max was of 0.55 ± 1.0 (range 0–2.9).

**Figure 1:**
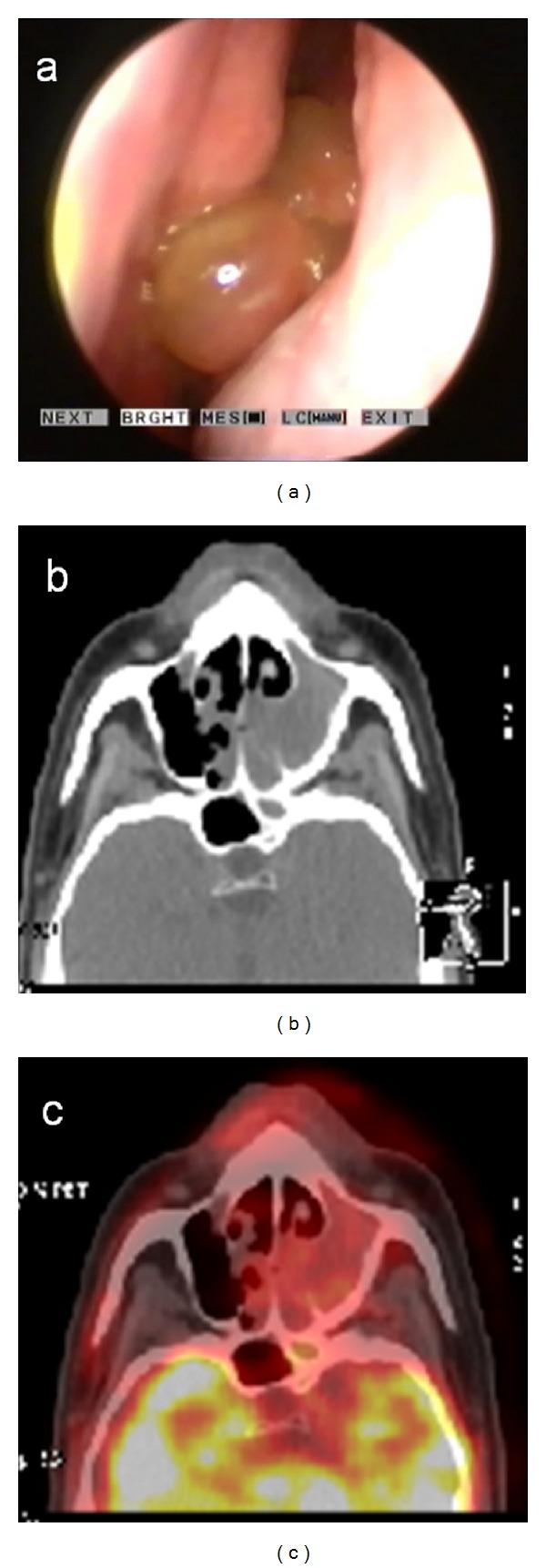
Case  7: (a) endoscopic view, (b) CT, and (c) ^18^FDG-PET/CT findings (SUV_max⁡_ 2.9).

**Figure 2:**
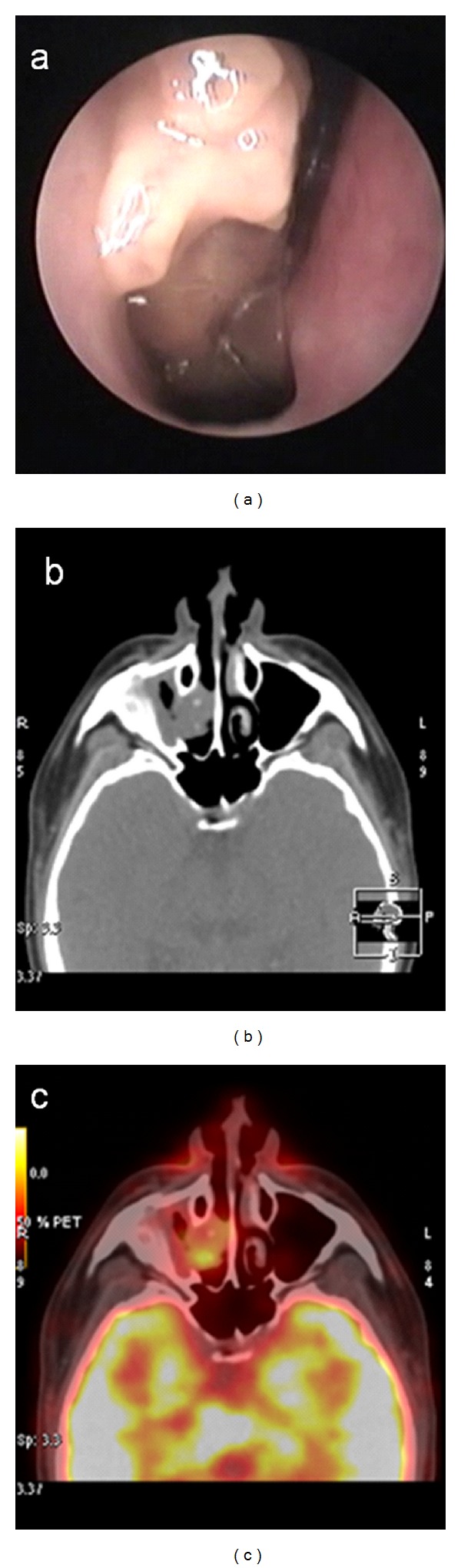
Case  11: (a) endoscopic view, (b) CT, and (c) ^18^FDG-PET/CT findings (SUV_max⁡_ 5.6).

The difference in the values of SUV_max⁡_ between patients with diagnosis of IP and negative for IP was statistically significant (*P* = 0.0001). Comparing FDG-PET/CT findings with histological diagnosis, FDG-PET/CT revealed a sensitivity and specify of 100%, considering a SUV_max⁡_ of <3.0 as a criterion for negative finding. Furthermore we noted that the cases with SUV_max⁡_ < 3 showed diffuse ^18^FDG uptake (Figure 1), while well-defined areas of ^18^FDG uptake (Figure 2) could be observed in the cases with SUV_max⁡_ > 3. 

## 4. Discussion

The literature on the diagnostic role of ^18^FDG-PET/CT in sinonasal IP remains scarce [8–12] and includes mostly case reports [8, 9]. In all reported cases, the ^18^FDG SUV_max⁡_ values for benign IPs were lower than those detected for IP with coexistent SCC. Ninomiya et al. [11] made a comparative study using C-choline and ^18^FDG PET on 22 patients with different pathological diagnosis of the nasal cavity and paranasal sinuses. This study include 5 cases of inverted papilloma with SUV_max⁡_ ranging from 2.30 to 5.20. In another original study Shojaku et al. [12] report five untreated patients with histological diagnosis of IP, made by biopsy in two different institutions, in which they perform a ^18^FDG-PET before surgical treatment. They found a SUV_max⁡_ ranging from 4.9 to 7.3 in 3 patients with IP and 8.9 to 20.9 in two patients with IP and SCC. In this our study, if we considered a cut-off of SUV_max⁡_ of >3.0, ^18^FDG-PET/CT was able to make diagnosis of IP with a sensitivity and specify of 100%. Lesions with a negative or diffuse ^18^FDG uptake with SUV_max⁡_ less than 3 should be considered negative for IP. A SUV_max⁡_ of <3.0 as a criterion for negative findings on FDG-PET has already been used by Yao et al. [13] in order to detect metastatic lymph nodes in head and neck tumors, reaching a sensitivity and negative predictive value of 100%.

In light of these data, we believe that with further investigations the range and the cut-off SUV_max⁡_ for the diagnosis of IP could be assessed.

PET/CT seems to be a useful diagnostic tool providing a definition of the location of IP and of the extent of disease, with the advantage (compared with CT alone and MRI) of integrating morphological and metabolic data in one single session [14]. We believe that the standardization of the range of SUV_max⁡_ in each institution is of key importance in order to determine the reliability of PET/CT for the diagnosis of IP.

Our study had some limitations, which included the small size of the series and the lack of comparison with MRI findings.

## Figures and Tables

**Table 1:** ^18^FDG-PET findings and clinical data of the patients.

Case	Age (years)	Sex	Location	SUV_max⁡_	Histopathological diagnosis
1	45	M	Nasal cavity, anterior ethmoid, maxillary sinus	6.1	Inverted papilloma
2	68	M	Nasal cavity, maxillary sinus	8.1	Inverted papilloma with associated foci of SCC
3	67	M	Nasal cavity	4.5	Inverted papilloma
4	73	M	Nasal cavity	—	Chronic inflammatory disease with a polypoid component
5	35	M	Nasal cavity, maxillary sinus	—	Hyperplastic mucosa with polypoid component
6	51	M	Nasal cavity, anterior ethmoid and maxillary sinus	—	Hyperplastic mucosa with massive polypoid component
7	65	M	Nasal cavity,maxillary sinus	2.9	Hyperplastic mucosa with massive polypoid component
8	58	F	Nasal cavity, anterior ethmoid and maxillary sinus	1	Hyperplastic mucosa with massive polypoid component
9	48	M	Nasal cavity, maxillary sinus	5.1	Inverted papilloma
10	62	M	Maxillary sinus	—	Hyperplastic mucosa with massive polypoid component
11	58	M	Nasal cavity	5.6	Inverted papilloma
12	72	M	Nasal cavity, maxillary sinus	—	Chronic inflammatory disease with a polypoid component
